# Large vesicles from ageing neutrophils: novel safeguards for the resolution of inflammation

**DOI:** 10.1038/s41392-025-02244-5

**Published:** 2025-05-19

**Authors:** Christopher Dietz-Fricke, Florian R. Greten

**Affiliations:** 1 Institute for Tumor Biology and Experimental Therapy, Georg-Speyer-Haus, Frankfurt/Main, Germany; 2 Department of Gastroenterology, Hepatology, Infectious Diseases and Endocrinology, Hannover Medical School, Hannover, Germany; 3 Frankfurt Cancer Institute, Goethe University Frankfurt, Frankfurt/Main, Germany; 4 German Cancer Consortium (DKTK) and German Cancer Research Center (DKFZ), Heidelberg, Germany

**Keywords:** Immunology, Cell biology

In a recent publication in *Cell*, Hsu and colleagues describe a novel kind of extracellular vesicle that is involved in the resolution of inflammation.^1^ Such large ageing neutrophil-derived vesicles (LAND-Vs) that express CD55^+^ and CD47^+^ are protected against efferocytosis and exhibit anti-inflammatory functions by inhibiting the complement activation. These findings reveal an important functional switch of neutrophils throughout the course of inflammation and identify a novel class of EVs that mediate neutrophil action beyond their limited lifespan, which may open new modes of therapeutic interference in various inflammatory conditions.

Neutrophils are one cornerstone of the innate immune response to pathogens.^2^ Neutrophils are equipped with a range of pattern recognition receptors and opsonin-mediated receptors, allowing for efficient sensing of pathogens. Once activated, neutrophils exhibit their antimicrobial properties by phagocytosis, release of toxic compounds including *reactive oxygen species* and myeloperoxidase. Besides direct antimicrobial functions, neutrophils also engage the adaptive immune response. Antigen presentation is enhanced through the recruitment of dendritic cells and direct antigen presentation to T- and B-cells, also increasing antibody production. While neutrophils are typically short-lived, neutrophil extracellular traps (NET) exhibit antimicrobial functions even after the original neutrophil died, thereby extending the temporal range of neutrophilic action.

While the immediate pro-inflammatory function of neutrophils during infection is well characterized, much less is known about their ability to counterbalance strong pro-inflammatory stimuli. Generally, neutrophil action is terminated and cleared by efferocytosis, the uptake of apoptotic neutrophils without the release of pro-inflammatory compounds. Further mechanisms skewing inflammatory processes driven by neutrophils towards resolution include resolvins, protectins, and maresins, summarized as *specialized pro-resolving mediators*.^3^ Derived from fatty acids, these mediators are also synthesized in neutrophils, limit neutrophil infiltration, and increase efferocytosis. In addition, the short lifespan per se is an additional limiting factor. Contrasting this, Hsu and colleagues now describe that ageing neutrophils in particular exhibit anti-inflammatory properties via the release of large vesicles.

These large-ageing-neutrophil-derived vesicles, short LAND-Vs, are greater than 1 µm of size and distinct from other vesicular structures such as apoptotic bodies or exosomes. LAND-Vs are predominantly and actively produced by ageing neutrophils but not apoptotic neutrophils. In a mouse-model of *Staphylococcus aureus* neutrophilic pneumonia, the presence of LAND-Vs led to a decline of infiltrating CD45^+^ cells, hinting towards a potential anti-inflammatory role of LAND-Vs. A more detailed characterization of LAND-Vs revealed an abundant CD55^+^ population among these vesicles. Given the known inhibitory effect of CD55 on complement activation, it was hypothesized that this particular CD55^+^ vesicle population exhibits an anti-inflammatory effect. This could be confirmed employing both CD55-deficient neutrophils as well as C3 knockout mice lacking a functional complement system. Specifically, CD55^+^ LAND-Vs inhibit the activity of the C3-convertase through direct binding to C3b/C4b, thereby preventing further complement activation. Blocking of CD55 inhibited the binding of LAND-Vs to C3b/C4b and reversed the anti-inflammatory phenotype. CD55 polarizes on the cellular membrane as neutrophils age, which is the site where LAND-Vs were being formed in an active process involving lipid rafts that required the activity of the actin cytoskeleton. Moreover, additional CD47 expression on LAND-Vs provides a “do not eat me”-signal thus limiting efferocytosis of these vesicles in the inflamed microenvironment. The anti-inflammatory properties could then be demonstrated in a model of lethal *Staphylococcus aureus* pneumonia where treatment with LAND-Vs led to improved survival rates. Interestingly, plasma samples from COVID-19 patients indicated an association of high levels of CD55^+^ CD47^+^ LAND-Vs with favorable clinical outcomes, suggesting an important contribution of such vesicles to the resolution of acute neutrophil-driven inflammation in human disease.

Neutrophils also play an important role in tumor development. Recently, prolonged neutrophil survival has been described in lung cancer due to tumor cell-derived GM-CSF.^4^ It will be interesting to examine whether CD55^+^ CD47^+^ LAND-Vs may as well contribute to the tumor-promoting function of aging neutrophils in the tumor microenvironment, as it has recently been shown for example for NETs. However, given the potential tumor-promoting effects of complement activation, CD55^+^ LAND-Vs could also be of therapeutic value in modulating the tumor microenvironment.

Research on therapeutic applications of extracellular vesicles is emerging. While EVs might score with longer shelf life and a favorable risk profile than replicating cell-based therapies, specific challenges are encountered.^5^ Besides the need for an in-depth understanding of the interplay with different tissues and cell types, more specific challenges include the optimization of circulation kinetics and targeting, with the potential need for engineered EVs. The detailed characterization of vesicle sub-populations and the subsequent functional validation conducted by Hsu and colleagues can serve as a starting point for translational investigations.

**Fig. 1:**
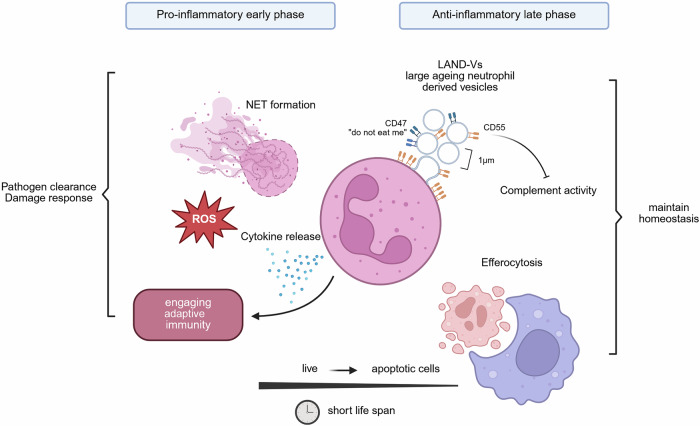
Neutrophils can have both pro- and anti-inflammatory functions. During an acute inflammation early on neutrophils contribute to a pro-inflammatory and potentially tissue damaging milieu by releasing cytokines, reactive oxygen species (ROS) and neutrophil extracellular traps (NETs). At later stages, in addition to efferocytosis, by generating large ageing neutrophil-derived vesicles (LAND-Vs) that express CD55^+^ and CD47^+^, neutrophils actively contribute to the resolution of inflammation even beyond their limited life span thereby ensuring proper dampening of inflammation required for tissue regeneration. Created with BioRender.com

In summary, Hsu and colleagues discovered a novel type of neutrophil-derived vesicles named LAND-V that is distinct from other EVs. The population of CD55^+^ CD47^+^ LAND-Vs is protected from efferocytosis and inhibits complement activation, thereby supporting inflammation resolution (Fig. 1). The association of LAND-Vs with favorable disease outcomes in a cohort of COVID-19 patients makes the prospective evaluation of LAND-Vs in acute infectious diseases promising. Ultimately, the observed survival benefit in an otherwise lethal mouse model of pneumonia further demonstrated the potential for therapeutic exploitation of CD55^+^ LAND-Vs.
